# Ovarian pregnancy: a case report in a resource-poor setting

**DOI:** 10.11604/pamj.2013.16.143.3505

**Published:** 2013-12-18

**Authors:** Florent Ymele Fouelifack, Jovanny Tsuala Fouogue, Jeanne Hortence Fouedjio, Zacharie Sando

**Affiliations:** 1 Obstetrics and Gynecology Unit of the Yaounde Central Hospital, Cameroon; 2 Faculty of Medicine and Biomedical Sciences of the University of Yaounde 1, Cameroon; 3 Pathology Unit of the Yaounde Gyneco-Obstetric and Pediatric Hospital, Cameroon

**Keywords:** Ovarian pregnancy, ectopic, Cameroon, ovary

## Abstract

Ovarian pregnancy is very rare and to our knowledge, no case has been reported in Cameroon. We herein report a case at the Yaounde Central Hospital. It is the case of a 29 years old woman who consulted in emergency for left pelvic pain at 9 weeks of pregnancy. The level of beta human chorionic gonadotropin was 96702 milli-international Units/ milliliter and ultrasound revealed an intra-ovarian gestational sac, an empty uterus and no peritoneal effusion. In the absence of facilities for laparoscopy, an emergency laparotomy was done. We found the non ruptured mass inside the left ovary. The left fallopian tube, the uterus and the right adnexae were normal. We did a successful ovarian dissection and resection of gestational sac. Trophoblastic tissue was found at pathology. Similar symptoms should draw attention of practitioners on the plausibility of ovarian pregnancy.

## Introduction

Ovarian pregnancy (OP) is characterized by implantation and evolution of the fertilized ovum on an ovary. Its pathophysiology is not fully understood. This entity is very rare and accounts for 0.5 to 3% of all ectopic pregnancies and 1/1543 to1/7000 of all live births [1, 2]. To our knowledge, no case has been reported in Cameroon. We herein present a case managed at the Yaounde Central Hospital.

## Patient and observation

**Figure 1:**
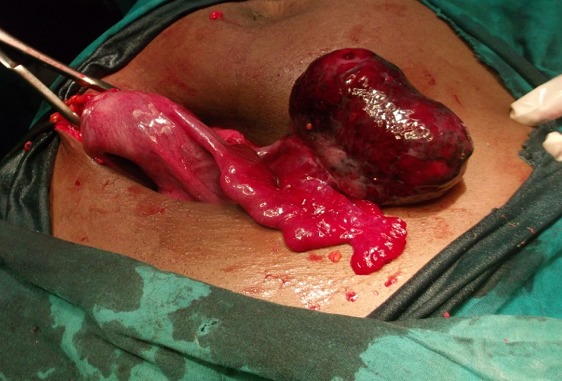
Picture showing the ovary containing the gestational sac, the uterus, the left round ligament, and the left Fallopian tube

**Figure 2:**
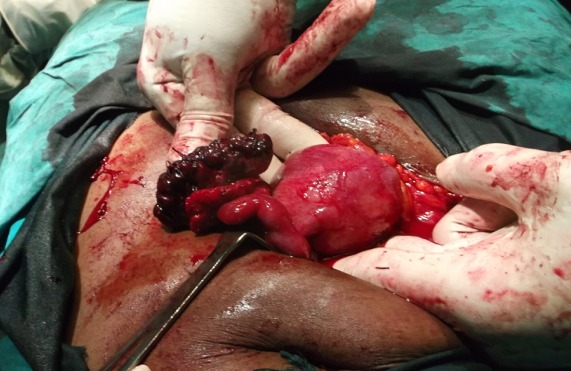
Operative aspect of the left ovary after resection and ablation of the gestational sac and hemostatic suture

**Figure 3:**
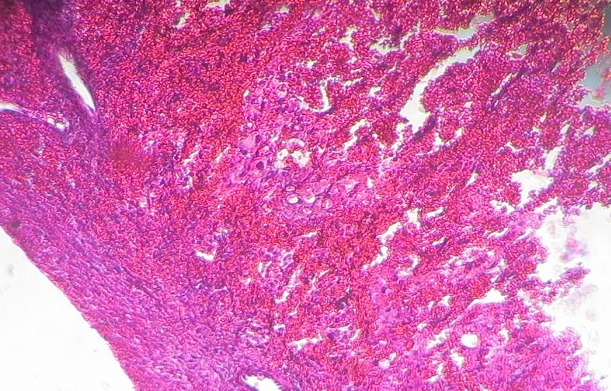
Micrograph of the specimen of ovarian resection under Hematoxylin-Eosin stain showing decidual cells, trophoblastic elements and the ovarian capsule

It is the case of a 29 years old woman, G2P1001, married, at 9 weeks of pregnancy. She consulted at our emergency service for the exacerbation of a left pelvic pain that has been evolving for two weeks prior to admission. A transient lull was observed following a treatment with a progesteron (hydroxyprogesterone caproate) and an antispasmodic (tiemonium methylsulfate) in a community clinic. The resurgence of that lancinating and permanent pain irradiating to the loins and associated with slight vaginal bleeding prompted consultation. She had first menses at 14 and her menstrual cycle is regular with a length of 30 days. She has never practiced contraception. She has been adequately treated for acute pelvic inflammatory disease five years earlier. Her only child is a girl born vaginally seven years ago. Her blood group is B rhesus positive and she has never undergone surgery. She presented with sympathetic signs of pregnancy and the urinary pregnancy test was positive but echography was not yet done. On admission, besides the main complain, the patient had vaginal bleeding and nausea but neither fever, nor vomiting. On physical examination her general condition was good and the blood pressure was 110/70 millimeters of mercury, the respiratory rate: 20 cycles / minute; the pulse rate: 70 pulsations/minute and the temperature: 37.3 degree Celsius. The conjunctivae were pink and the tongue was clean and moist. There were no cervical adenopathies. The breast and cardiopulmonary examination revealed no abnormalities. The abdomen was flat, and mobile with respiration. On palpation there was just a tenderness of the left iliac fossa and the bowel sounds were normal on auscultation. Inspection under speculum revealed normal gravid external cervical os and there was no vaginal discharge. On digital exploration, the cervix was posterior long and closed, the uterus was globular, increased in size and compatible with an eight weeks pregnancy. The left adnexae presented with a tender, smooth and mobile mass of seven centimeters diameter but the right one were normal. The posterior cervico-vaginal fornix was neither tender nor bulging. We suspected a non ruptured extra-uterine pregnancy with the following differentials: heterotopic pregnancy, torsion of ovarian cyst in pregnancy, and intra-cystic ovarian bleeding in pregnancy. Paraclinical investigations revealed: beta human Chorionic Gonadotropin (β hCG) level of 96 702 milli International Units per milliliter of plasma (mIU/ml). Ultrasonography revealed a heterogenous left ovarian mass of 82 millimeters in diameter, an empty uterus and no peritoneal effusion. This comforted our first diagnosis. After a normal pre-operative work up, an emergency laparotomy was done under general anesthesia. The findings were: left ovary containing a gestational sac and the corpus luteum, normal right adnexae, normal left tube and normal uterus (Figure 1). There was no hemoperitoneum. We dissected the ovarian capsule and carried out ablation of the gestational sac, then we did hemostasis (Figure 2). The specimen was analysed by the pathologist who found decidual cells and trophoblastic tissue within the ovarian capsule and thus confirmed the ovarian pregnancy (Figure 3). Post operative course was uneventful and the patient was discharged six days after surgery. The βhCG level decreased and disappeared 25 days after surgery.

## Discussion

The mean age at diagnosis of OP is 29 years [1], which is the age of our patient. The commonly described profile of women presenting with OP is pauciparity with no history of infertility [1–3]. This is in conformity with our patient. Indeed tubal defects and subsequent infertility are known risk factors for other ectopic pregnancy but not for OP [3]. Intra-uterine contraceptive device (IUCD) is found in up to 68% of OP and its frequency is also higher in case of endometriosis, assisted reproductive technology and emergency contraception [3]. Fallopian tube reflux is today the most commonly admitted pathophysiological mechanism leading to OP in the presence of IUCD [3]. Alteration of the tubal ciliary function due to IUCD is also evoked as a possible mechanism [2, 4–5]. Our patient did not have any of the preceding risk factors.

Several clinical forms of OP have been described: intra-ovarian gestational sac with or without embryo, ruptured gestational sac with hemoperitoneum, molar pregnancy, and twin pregnancy [5]. In this case we observed an ovarian non ruptured gestational sac without embryo. Our patient presented with left pelvic pain at nine weeks of pregnancy without signs of hemodynamic instability. The emptiness of the uterus and the presence of an ovarian mass comforted the diagnosis. Our patient presented metrorrhagia as in 67% of cases in literature [5]. Typical ultrasound findings include: emptiness of the uterus, rosette-like image of the ovary containing a gestational sac with or without embryo and eventually peritoneal effusion. The preceding signs, when associated to a β hCG level higher than 1500mIU/ml, highly suggest the diagnosis [3]. Rupture of OP classically occurs during first trimester and is preceded by pain corresponding to distension of the ovarian capsule. In developed countries, due to early diagnosis, only 8 to 21% of OP are observed with hemodynamic shock [4]. The Spielberg's diagnostic criteria defined in 1878 are still used and they were respected in our case [3, 5]. Indeed, pathologists found trophoblastic tissue and decidual cells within the ovary, the gestational sac was irrigated by the ovarian artery and the homolateral tube was macroscopically normal.

Laparoscopy is the cornerstone of modern management of OP. This technique is dependent on sophisticated equipment, surgical skills, and earliness of diagnosis. Thus it is not always available in ressource-poor settings [5]. For lack of equipment and skills adapted to laparoscopy we carried out an emergency laparotomy. The state of the concerned ovary allowed us to perform a dissection and resection of the gestational sac. In some cases where the ovary is much altered, total oophorectomy may be done. Antimitotics drugs like methotrexate are added when the decrease of level of β-hCG is not sufficient [3]. We did not use it in this case because the drop in level of β-hCG was satisfactory.

## Conclusion

Illustration of this case of OP in our setting shall raise awareness on this rare disease in the mind of practitioners.
